# Can the digitalization level of agriculture improve its ecological efficiency under carbon constraints: Evidence from China

**DOI:** 10.1016/j.heliyon.2024.e26750

**Published:** 2024-03-02

**Authors:** Haoran Wang, Zhuangzhuang Li

**Affiliations:** a Research Centre for Urban and Rural Big Data Development and Digital Governance, Suzhou University, Suzhou, 234000, China; b JingDian Digital and Data Intelligence Research Centre, Anhui JingDian Market Research and Consulting Co., Ltd., Hefei, 230031, China; c Financial and Statistical Analysis Research Centre, Suzhou University, Suzhou, 234000, China

**Keywords:** Agricultural ecological efficiency, Carbon emission, Agricultural digital level, Moran index, Spatial effect

## Abstract

The interplay between digitalization and economic development constitutes a pivotal global issue, yet empirical research on agricultural ecological efficiency in developing countries remains limited. This study initially establishes a measurement system and a comprehensive index for the level of agricultural digitalization. Subsequently, it delineates the relationship between agricultural digitalization level and agroecological efficiency using the spatial Durbin model, and ultimately explores the enhancing effect of agricultural digitalization level on agroecological efficiency using China as a case study. Research reveals that the agricultural ecological efficiency across the 31 mainland Chinese provinces demonstrates a generally linear upward trajectory, embodying both agglomeration and heterogeneity. The level of agricultural digitization exerts a significant, positive direct impact and facilitates a spatial spillover effect on agricultural ecological efficiency. Other control variables, such as financial support for agriculture and local economic development, impart a positive direct impact on regional agricultural ecological efficiency, while rural household operating income propels a positive spatial spillover effect on adjacent areas. The findings furnish guidance for developing countries to adeptly execute digital rural construction, aiming to enhance agricultural ecological efficiency amidst carbon constraints.

## 1 Introduction

Agroecological efficiency refers to the pursuit of maximizing agricultural output while minimizing resource use and environmental damage in the agricultural production process. Improving agroecological efficiency is an important issue in the sustainable development of agriculture, and realizing the improvement of agroecological efficiency under environmental constraints, in particular, is one of the important goals of governments around the world [1]. China, being a major agricultural producer and the world's most populous country, not only seeks to augment its agricultural output but also enhance product quality. The Chinese government has fortified protections for agricultural land, stringently prohibiting encroachments, while simultaneously accelerating new urbanization and implementing measures to bolster the country's agricultural output. From 2010 to 2020, China witnessed a 1.19-fold increase in grain output, including rice and wheat, which respectively experienced 1.07 times and 1.16 times growth, thereby solidifying China's self-sufficiency in staple foods. Nevertheless, the adoption of petroleum agriculture has precipitated “reverse ecological” consequences, such as agricultural nonpoint source pollution, soil fertility degradation, and resource wastage [2,3]. Therefore, investigating strategies to enhance agricultural ecological efficiency while ensuring consistent growth in agricultural output has surfaced as a significant issue both in China and globally. Amidst the swift advancement of the information industry, epitomized by big data, cloud computing, and blockchain, digital technology has increasingly permeated various sectors of China's economy and society. Elevating China's agricultural ecological efficiency is intrinsically tied to the development of digital rural areas and agriculture. Regrettably, existing literature predominantly explores agroecological efficiency influencers from the perspectives of traditional factor input and social development [4], and rarely studies the impact of the agricultural digitalization level on agroecological efficiency from the perspective of the digital economy, which provides an opportunity to pursue this research.

Given the aforementioned considerations and utilizing statistical yearbook data from 31 Chinese mainland provinces from 2010 to 2020, this paper focuses on the planting industry. It employs the Super-SBM model and systematic clustering method to measure agricultural ecological efficiency and its spatial heterogeneity, and evaluates the spillover effects of influencing factors using the spatial Durbin model, with the agricultural digitization level as the core explanatory variable. This paper offers marginal contributions in four primary dimensions: Firstly, while existing studies on digital level predominantly measure economic or regional global digital levels, this research constructs a digital level measurement system and a comprehensive agricultural index, thus augmenting the digital level measurement's content system. Secondly, unlike prior works that only consider the expected gross agricultural output when measuring agroecological efficiency, this paper also accounts for the undesired output of agricultural carbon emissions, thereby enriching the agricultural ecological efficiency measurement. Thirdly, while past studies on China's agricultural ecological efficiency typically rely on administrative boundaries and treat study subjects as separate spatial units, neglecting unit correlation, this paper utilizes a space 0–1 matrix as the weight. The spatial global Moran index and the local Moran index further unveil the spillover effect between units, contributing to research on agriculture's high-quality, integrated development. Fourthly, in terms of researching the influencing factors of agricultural ecological efficiency, this paper goes beyond examining macroeconomic policies, farmer development, and agricultural resource element input, which is the primary focus in existing literature, by also considering the novel element of data resources. The direct and indirect effects of agricultural digitization on China's agricultural ecological efficiency have been empirically tested using the spatial Durbin model, thereby enriching conclusions about the relationship between digital technology and agricultural ecological efficiency.

The remainder of this paper is organized as follows: Section two provides a literature review, while section three offers theoretical analysis, research methods, and data, which inform the design of research materials and construction of an index system. Section four delivers an analysis of the empirical results, encapsulating measurement results of China's agricultural ecological efficiency, spatial heterogeneity analysis, influencing factor analysis, and robustness analysis. The final section details research conclusions, policy guidance, research deficiencies, and recommends avenues for subsequent work.

## 2 Literature review

The concept of agroecological efficiency is derived from the concept of “ecological efficiency” proposed by Schaltegger and Sturm in 1990, and it is mainly used to reflect the quantitative relationship between agricultural input and output. Historically, prominent methodologies applied in agroecological efficiency research encompass the ratio method, life cycle method, stochastic front method (SFA), ecological footprint method, and data envelope analysis method (DEA). Research evidence that each method carries its own merits and drawbacks [5]. Notably, SFA and DEA have emerged as the most prevalent approaches, especially DEA, which is lauded for its advantages in multi-index macro-evaluation. The method facilitates the quantitative measurement of agricultural production efficiency according to a predefined function during the specific evaluation process, mitigating bias from the subjective weighting of multiple indicators and gaining wide acknowledgment in academia [6]. In 2001, Tone added the relaxation variable to the target function, which further improved the accuracy of the agricultural ecological efficiency measurement in the model. The DEA-SBM model has progressively evolved, accommodating more inputs and outputs than its mainstream counterparts, and has been extensively utilized by scholars assessing agricultural ecological efficiency in China.

Developed countries study agricultural ecological efficiency with a focus on fairness and efficiency, research the maximization of well-being with minimal environmental loss, and propose policy suggestions from the perspective of improving the utilization efficiency of agricultural resources [[7], [8], [9]]. Current domestic research evaluating China's agricultural ecological efficiency primarily pivots on economic theory, exploring ways to maximize the value of the Chinese agricultural economy from a sustainability standpoint [10,11]. The research content focuses on the measurement of agricultural ecological efficiency in different periods within the same space, and the differences are mainly reflected in the selection of input indicators and output indicators [4,12]. Most scholars have found great differences in agricultural ecological efficiency among different regions [13,14], and agricultural ecological efficiency in the same region shows different varying trends across different periods [15,16]. Liu et al. [17] measured the ecological efficiency of broad agriculture in China from 1978 to 2017 and found that the agroecological efficiency increased from 0.405 in 1978 to 0.713 in 2017, a 76% increase, though accompanied by significant variations across different regions. The agricultural ecological efficiency in Northeast, East, and South China is higher than the national average. The Southwest and Northwest regions are both below the national average. Yang et al. [15] evaluated the ecological efficiency of cultivated land utilization in the Yangtze River Economic Belt from 2001 to 2018. The results showed a decrease in ecological efficiency values from 2001 to 2004, which showed a rapid upward trend from 2005 to 2018. The highest efficient region is located in the lower reaches of the Yangtze River. The findings indicate a markedly different space-time effect of farmland utilization ecological efficiency in the Yangtze River Economic Belt as compared to other regions. Furthermore, several scholars have explored conventional factors influencing the regular fluctuation trend of agricultural ecological efficiency in China. Zhao et al. [18], after measuring land ecological efficiency in the Beijing-Tianjin-Hebei region from 2005 to 2013, quantified the impact on land ecological efficiency with the new urbanization level as the core, revealing an “N"-shaped curve relationship between land ecological efficiency and new urbanization. This indicates that, when considering the development of the new type of urbanization, land ecological efficiency exhibits a trend of initial growth, subsequent decline, and eventual resurgence. Han et al. [19] investigated the influence of management efficiency and technological level on the variance in agricultural ecological efficiency in 30 Chinese provinces from 1997 to 2015 from management and technology perspectives, deducing that the nationally observed low agroecological efficiency originates from a blend of suboptimal management efficiency and an inefficient technological gap. At the regional level, the depressed agroecological efficiency in northeast China is wholly attributed to deficient management efficiency, while the reduced agricultural ecological efficiency in central and western regions results from both low management efficiency and an inferior technological gap efficiency.

Upon reviewing the existing literature, it is apparent that current research reveals three key gaps. Firstly, in computing China's agricultural ecological efficiency, a majority of studies utilize gross agricultural output value as an output index, employing inputs such as labor, land, water resources, mechanical power, agricultural fertilizer, and plastic film to explore agricultural total factor productivity amidst resource constraints. This approach overlooks the impact of national financial input and undesired outputs on agroecological efficiency. Secondly, prevalent studies tend to center on the direct impact of various factors on agricultural ecological efficiency, such as the economic development level, urbanization rate, agricultural planting structure, scientific and technological innovation, and degree of mechanization, while neglecting the often-present spatial relevance of agroecological efficiency. Existing studies seldom probe into the spatial spillover effects of these influencing factors. Thirdly, despite the notable positive influence of the rapidly advancing digital technology across all domains, there is a palpable scarcity in the literature examining the impact of the level of agricultural digitalization on agricultural ecological efficiency, especially in the context of vigorously advancing the digitalization of agriculture and rural areas. This leaves a noticeable gap in the research related to the level of agricultural digitalization. Consequently, considering the aforementioned deficiencies and establishing an agricultural digital level measurement index system, this research aims to gauge the spatial and temporal fluctuations in China's agricultural ecological efficiency and to determine its influencing factors.

## 3 Theory, methods, and data

### 3.1 Theoretical analysis

“High-quality development,” “common prosperity,” and reaching a “carbon peak” stand as the three pivotal goals presently steering the Chinese government's agenda and will continue to do so in the foreseeable future. Among them, high-quality development is embodied in the combination of “quality” and “efficiency.” On the premise of not damaging the ecological environment, innovation should promote quality change, efficiency change, and dynamic change in economic development [20,21]. Agricultural high-quality development, pivotal to this overarching aim, not only forms a crucial facet of high-quality development but also underpins it [22]. Conversely, common prosperity emerges as a pivotal goal of the CPC Central Committee, aspiring to enhance the well-being of the populace after achieving poverty alleviation. The complexity of actualizing this goal is accentuated in rural contexts, with a crucial focus on agriculture. High-quality agricultural development can substantively boost farmers' incomes. Achieving a carbon peak is one of the “dual carbon” goals set by the Chinese government. With commitments to attain a carbon peak and “carbon neutrality” by 2030, and considering agriculture as a substantial carbon emission source, second only to the industrial sector [23,24], China, as a preeminent agricultural nation, faces a long journey towards reducing agricultural carbon emissions. Agricultural ecological efficiency, being a pivotal indicator of high-quality agricultural development, necessitates enhancing such efficiency towards low-carbon development. This is not only imperative for realizing high-quality agricultural development but also emerges as a critical pathway towards augmenting farmers' incomes.

Regarding the analysis of the influencing factors of agroecological efficiency, scholars at home and abroad have already tested in detail the conduction mechanism and the size of the role of traditional factors such as the level of regional economic development, financial support for agriculture and the degree of agricultural mechanization on agroecological efficiency [25], but digital technology, as a kind of advanced informatization technology, lacks a theoretical analysis and a mechanism test of the conduction path of agroecological efficiency. Based on the findings of existing scholars [26,27], this paper argues that agricultural digital technology will have an impact on agroecological efficiency through the following four paths.

First, it will have a positive impact on agroecological efficiency through the production system, and the digital technology can help agricultural producers realize the precise and intelligent management of agricultural production. For example, remote sensing technology and GPS technology can help agricultural producers realize precise fertilization, precise control of pests and diseases, reducing the damage of fertilizers to the agricultural ecosystem while improving crop yield and quality; through the Internet of Things technology to help agricultural producers realize intelligent irrigation, saving water resources while replenishing water to crops at the right time to improve the use of water resources for crop growth efficiency; through smart sensors and digital Through intelligent sensors and digital twin technology, real-time monitoring of crop growth and growth environment can help agricultural producers adjust planting structure and production management in time, quickly obtain solutions and improve agricultural production efficiency.

Secondly, through the positive impact on agro-ecological efficiency of the operating system, digital technology can grow the scale of agricultural operators and innovate agricultural business models. For example, the application of agricultural big data, Internet of Things and Internet technology can help agricultural producers expand the scale of agricultural cultivation, optimize the structure of agricultural planting according to consumer demand, and realize intensive land production and management, which not only reduces the cost of production from the input side, but also extends the industrial chain to produce agricultural products more in line with the market demand from the output side, and some new agricultural operators who know the technology and know how to manage continue to increase the overall efficiency of agriculture and improve agricultural production through the innovation of agricultural operation mode. Some new agricultural operators who know technology and know how to manage continuously improve the total factor productivity of agriculture through innovative agricultural business models, which helps to promote the improvement of agro-ecological efficiency.

Thirdly, the circulation system has a positive impact on agro-ecological efficiency, and digital technology can realize the diversification of agricultural supply channels and the speed of supply. For example, through the agricultural production and trading platform can real-time control of a variety of agricultural products, such as regional distribution, maturity time, total supply and consumer demand characteristics of agricultural products, such as production, exchange of agricultural products, the whole process of information, after the analysis of big data can be quickly guide the agricultural products transportation network layout and transportation mode, so that the circulation of agricultural products in the supply and demand for more efficient, effective shortening of agricultural products, saving the time of storage This effectively shortens the storage time of agricultural products, saves the inventory cost of agricultural products, reduces the value loss of agricultural products, and improves the safety and quality of agricultural products.

Fourthly, through the consumption system, it has a positive impact on agroecological efficiency, and digital technology helps consumers buy agricultural products that offer good value for money. Digital technology has given rise to a new model of live video sales, through live video sales can help consumers see the whole process of agricultural production, individual consumers can subscribe to a cow, subscribe to a tree, or even subscribe to a piece of land, from a seed into a mature fruit consumers participate in the whole process, the higher the degree of consumer participation in the business process of agricultural products, the more able to increase the added value of agricultural products, and at the same time, the more able to reduce the value of agricultural products in the production and management process, the more the consumer is able to reduce the value of agricultural products in the production and management process. The higher the degree of consumer participation in the agricultural business process, the more it can increase the added value of agricultural products, and at the same time, the more it can reduce the loss of value in the production and management process of agricultural products and the destruction of resources and environment.

**Fig. 1:**
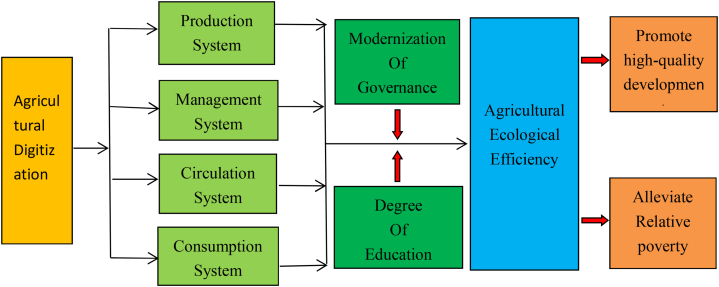
Mechanism of agricultural digitalization technology affecting agricultural eco-efficiency.

In conclusion, agricultural digital technology can have a positive impact on agricultural ecological efficiency through the four paths of production system, operation system, circulation system and consumption system. In addition, the improvement of the education level of agricultural operators contributes to the application of digital technology in the field of agricultural production and management, and the enhancement of the level of modernized governance also contributes to the contribution of digital elements to agricultural production and management, and the level of education and modernized governance strengthens the impact of agricultural digital technology on agricultural ecological efficiency. The role of agricultural digital technology on agroecological efficiency, which in turn promotes the high-quality development of agriculture and the reduction of relative poverty of farmers, the path of the role of agricultural digital technology on agroecological efficiency can be succinctly expressed as shown in Fig. 1. Based on the above analysis, the research hypothesis 1 of this paper is proposed:

Hypothesis 1(H1) Agricultural digitalization technology can have a significant positive impact on agroecological efficiency.

This paper empirically tests the relationship between agricultural digital technology and agroecological efficiency using regional data in China as a case sample. Because Chinese agriculture is spatially vast, both agricultural digital technology and agroecological efficiency show imbalance in spatial performance, and the causal relationship between the two will be affected by the spatial distribution of the sample; in addition, agricultural digital technology consists of two parts, digital infrastructure and application level, and digital infrastructure construction can not only benefit the region, but also can bring the policy of neighboring regions dividends, so the research hypothesis 2 of this paper is proposed:

hypothesis 2(H2) The impact of agricultural digitalization technology on agroecological efficiency in this region has a spatial spillover effect.

### 3.2 Research method

#### 3.2.1 Super-SBM model

In order to test the relationship between digital technology and agroecological efficiency, it is first necessary to measure agroecological efficiency, taking Chinese agriculture as an example. In this paper, when measuring the agro-ecological efficiency in China, in addition to the introduction of the gross agricultural product in the output indicators, the carbon emissions in the agricultural production process (called non-desired outputs) are also added, so it is called the agro-ecological efficiency under carbon constraints. The traditional DEA model can only measure the value of agroecological efficiency (DMU) without non-desired outputs, while the SBM model can measure the DMU under the influence of non-desired outputs by introducing the non-desired output factor into the planning model, but the DMU value measured by the non-desired outputs SBM model is less than or equal to 1, which has a truncated tail, and it cannot be further utilized by spatial econometric models to conduct other correlation analyses on it. Compared with similar literature, this paper uses an improved non-expected output SBM model, called “Super-SBM”, to measure the agroecological efficiency in China, which not only solves the effect of non-expected output on the value of agroecological efficiency but also allows the calculated efficiency to exceed the value of DMU by the introduction of relaxation variables. Moreover, the calculated efficiency value is allowed to exceed 1, which solves the problem of not applying to spatial panel model. Consequently, this paper employs the Super-SBM model to measure the agricultural ecological efficiency across 31 provinces on the Chinese mainland. The specific model form is as follows:

$$DMU=\min\frac{1+\frac{1}{m}\sum_{i=1}^{m}\frac{\overset{x}{\underset{i}{s}}}{\overset{i0}{x}}}{1-\frac{1}{\overset{1}{s}+\overset{2}{s}}\left(\sum_{k=1}^{\overset{1}{s}}\frac{\overset{y}{\underset{k}{s}}}{\overset{k0}{y}}+\sum_{l=1}^{\overset{2}{s}}\frac{\overset{z}{\underset{l}{s}}}{\overset{l0}{z}}+\right)}$$

(1) $$\begin{array}{l} \begin{array}{cc} s.t. & x_{i0} \end{array}\geq \sum_{j=1}^{n}\lambda_{j}x_{j}-\begin{array}{cc} S_{i}^{x} & \forall i \end{array}\\ \begin{array}{cc} \; & y_{k0} \end{array}\leq \sum_{j=1}^{n}\lambda_{j}y_{j}-\begin{array}{cc} S_{k}^{y} & \forall k \end{array}\\ \begin{array}{cc} \; & z_{l0} \end{array}\geq \sum_{j=1}^{n}\lambda_{j}z_{j}-\begin{array}{cc} S_{l}^{z} & \forall l \end{array}\\ \begin{array}{cc} & 1-\frac{1}{\overset{1}{s}+\overset{2}{s}}\left(\sum_{k=1}^{s_{1}}\frac{S_{k}^{y}}{y_{k0}}+\sum_{l=1}^{s_{2}}\frac{S_{l}^{z}}{z_{l0}}+\right) \end{array}>0\\ \begin{array}{cc} & S_{i}^{x}\geq 0, \end{array}S_{k}^{y}\geq 0,S_{l}^{z}\geq 0,\lambda_{j}\geq 0,\forall i,j,k,l \end{array}$$

where *DMU* is the value of agroecological efficiency under carbon constraints, $x_{i0},y_{k0},z_{l0}$ represent the input index, expected output, and unexpected output, respectively, $s_{i}^{x},s_{k}^{y},s_{l}^{z}$ denote the input index, expected output, and unexpected output relaxation variables, respectively, and $\lambda_{j}$ represent the intensity variables.

#### 3.2.2 Space panel model

The Spatial Panel Lag Model (SPLM), Spatial Panel Error Model (SPEM), and Spatial Panel Durbin Model (SPDM) are three frequently utilized spatial panel regression models [28]. The SPDM, encapsulating certain advantages of both the SPLM and SPEM, is not only proficient in addressing the spatial correlation and heterogeneity of the variables under scrutiny but also adept at quantifying their direct and spatial spillover effects [29,30]. Due to its superior ability to handle spatial causality, the spatial panel Durbin model is preferred. This model has been chosen as the analytical framework for investigating the influencing factors of agricultural ecological efficiency in China:

(2) $$Y_{it}=\alpha X_{it}+\beta \sum_{j=1}^{N}W_{ij}Y_{it}+\gamma \sum_{j=1}^{N}W_{ij}X_{it}+\mu_{i}+\nu_{t}+\varepsilon_{it}$$

where *Y*_*it*_ and *X*_*it*_ are the observed values of the explained variable and explanatory variables in provinces *i* and *j* in year *t*, respectively, *W*_*ij*_ is the spatial weight matrix, α is the vector of the estimated parameter of the explanatory variable, β is the spatial lag coefficient of the explained variable, γ is the spatial regression parameter of the explanatory variable, $\mu_{i}$ and $\nu_{t}$ are the individual effect and time effect, respectively $\varepsilon_{it}$ is the random error term, and $\varepsilon_{it}∼N\left(0,\sigma^{2}\right)$. When γ=0,β≠0, Equation (2) degenerates into the SPLM, and when γ+αβ=0, Equation (2) degenerates into the SPEM. To increase the reliability of the model estimation results, this paper first takes the logarithm of the variable index, and then the two spatial weighting methods of the 0-1 adjacent matrix and geographic distance matrix are used to estimate the model. For the calculation methods of the two matrices, please refer to the literature [31].

### 3.3 Index system and data sources

#### 3.3.1 Indicator system

To select indicators to measure agricultural ecological efficiency, this paper refers to the literature [20,32], takes the narrow sense of agriculture (planting industry) as the research object, and constructs the Chinese agricultural ecological efficiency measurement index system from the following three aspects: input elements, expected output, and undesired output factors (see Table 1 for details). The input indicators comprise fixed assets, labor force, land, chemical fertilizers, pesticides, agricultural film, mechanical power and diesel oil, and the output indicators comprise expected output agricultural GDP and undesirable output agricultural carbon emissions. The calculation of agricultural carbon emissions follows the practice of Basit et al. [33] and weights the carbon emissions of agricultural material input and rice cultivation by using the carbon dioxide conversion coefficient provided in the fourth assessment report of the IPCC.

**Table 1:** Input-output index of agricultural eco-efficiency in China.

Main Index	Secondary Index	Variable Specification
Input index	Fixed assets (RMB 100 million)	Part of the investment is used for the purchase of equipment and tools
	Labor input (10 thousand people)	Number of agricultural employees
	Land input (thousand hectares)	Total sown area of crops
	Fertilizer input (10 thousand tons)	Conversion amount of agricultural fertilizer application
	Pesticide input (ton)	Pesticide usage
	Agricultural film input (ton)	Plastic film usage
	Mechanical power input (10 thousand kw)	Total power consumed by agricultural machinery
	Diesel input (10 thousand tons)	Agricultural diesel consumption
Expected output	Gross agricultural product (RMB 100 million)	Agricultural output value in the total output value of agriculture, forestry, animal husbandry and fishery
Undesirable output	Agricultural carbon emissions（10 thousand tons）	According to the carbon emission coefficient published by the IPCC

In determining the indicators for the core explanatory variable, the agricultural digitization level (DIGI), this paper initially consults the evaluation index system of agricultural and rural digital economy development constructed by Qi et al. [34]. The “rural digital industrialization” index, commonly utilized in these systems, is omitted chiefly because China's current agricultural digital industrialization has not yet achieved a substantial scale and exerts minimal impact on agroecological efficiency. Subsequently, we augment the “computer penetration rate” and “Taobao village proportion” as indicators. These represent the level of infrastructure development and the application of rural e-commerce, respectively, thereby addressing the shortcomings of the index system derived from Kelly et al. [35] (Further details are provided in Table 2).

**Table 2:** Index system of Chinese agricultural digitization level evaluation.

First-order index	Second-order index	Indicator description
Digital infrastructure	Rural Internet penetration rate (%)	The number of Internet users in rural areas accounts for the rural population in the area
	Smartphone penetration rate in rural areas (PCS)	Number of mobile phones owned by rural residents per 100 households per year
	Computer penetration rate in rural areas (PCS)	The average number of computers owned by rural residents per 100 households per year
	Radio and television network coverage rate (%)	The actual number of rural cable broadcast TV users accounts for the total number of rural households
	Agrometeorological observation station (PCS)	Number of agrometeorological observation stations
Digital application level	agricultural products (RMB 100 million)	Transaction volume of rural e-commerce
	Agricultural digitization scale (%)	The proportion of digital technology application scale in the planting industry
	Proportion of Taobao Village in all administrative villages (%)	The number of Taobao villages accounts for the total number of administrative villages in the region
	Consumption level of digital products and services (%)	The proportion of annual consumption expenditure of rural households using smart devices, software, and other digital life products and services
	Digital finance	Digital Financial Inclusion Index

Based on prior research [36,37], the selection of control variables typically includes factors like urbanization rate, industrialization level, regional economic development level, financial agriculture support, cultivated land scale intensity, proportion of rural households' operating income, per capita net income of rural residents, agricultural mechanization density, agricultural disaster rate, agricultural planting structure, and per capita agricultural added value. Considering that the agricultural digitization level of the comprehensive index measured in this study may have some degree of correlation with the above control variables, we employed the variance inflation factor (VIF) to address any multicollinearity concerns, ensuring the reliability of our regression analysis. Consequently, five indicators, namely regional economic development level (GDP), financial support for agriculture (FINA), rural household operating income ratio (POINC), agricultural mechanization density (AGMD), and agricultural disaster rate (AGDR) - are selected as control variables for estimating the regression model. The VIF values for the six variables, which include the core variable of agricultural digitization level, are all under 5, indicating no multicollinearity issue within the model.

#### 3.3.2 Data sources

Data for this research primarily originates from the China Statistical Yearbook, China Rural Statistical Yearbook, and various provincial and municipal Statistical Yearbooks. Additional index data are sourced from the Digital Inclusive Finance Index, published by Peking University's Digital Finance Research Centre, and research reports from the Ali Research Institute. For instances of missing data across specific provinces and years, interpolation was performed using average and average growth rate methods.

## 4 Empirical analysis

### 4.1 The evolution of the space-time effect of agricultural ecological efficiency in China

**Fig. 2:**
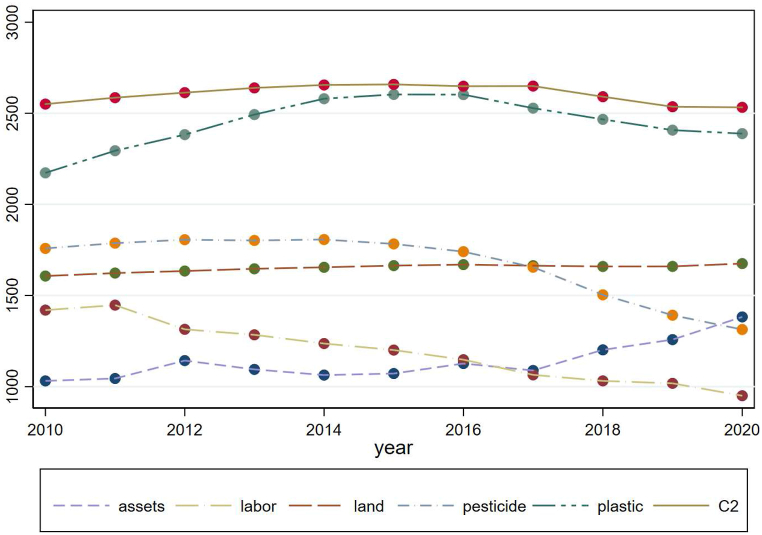
Change in trend of China's agricultural input-output from 2010 to 2020.

**Fig. 3:**
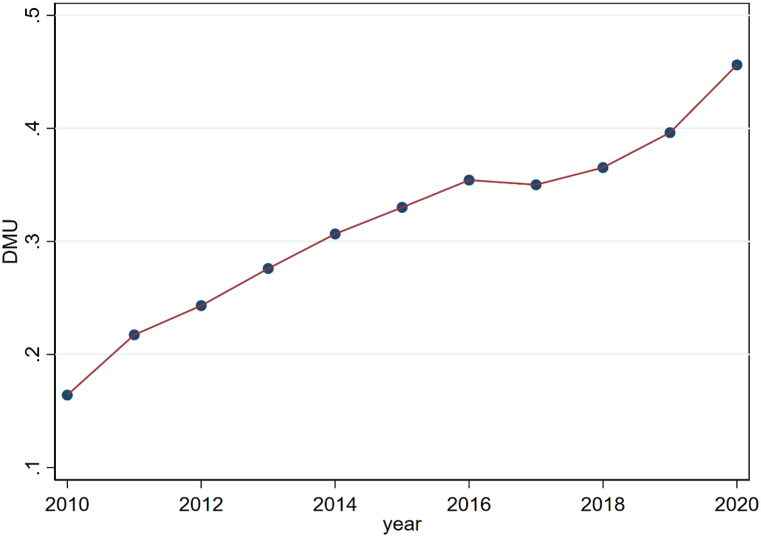
Change in trend of agricultural eco-efficiency in China during 2010–2020.

After establishing the model, setting the index system, gathering data, and using equation (1), this study initially examines the visual trends of China's agricultural input and output and agricultural ecological efficiency from 2010 to 2020, as shown in Fig. 2, Fig. 3. To further describe the provincial differences in China, the systematic clustering method (hierarchical clustering method) is employed to cluster the agricultural ecological efficiency of the 31 provinces in mainland China.^1^ Analysis indicates varying trends in the agricultural input-output index across China and significant disparities in the evolution of agricultural ecological efficiency among different provinces.

#### 4.1.1 Change in trend of agricultural input and output in China

Considering the overall shift in agricultural input factors, it is observed that while land and fixed assets demonstrated a rising trend from 2010 to 2020, labor input significantly receded. Contrastingly, the input of pesticides, fertilizers, and agricultural film, notable contributors to agricultural nonpoint source pollution, markedly descended after 2016, with agricultural carbon emissions from undesirable outputs also beginning to taper off after that year. Notably, in 2016, China proposed the goal of a carbon peak in the Paris Agreement. In October 2017, during the 19th National Congress of China, the nation introduced the concept of ecological and human harmony, championing the view that clear waters and green mountains are invaluable assets. This perspective has propelled China to tighten its carbon emission controls across various sectors, including agriculture, and to formulate more rigorous policies for safeguarding agricultural ecology.

#### 4.1.2 The trend change of agricultural ecological efficiency in China

Utilizing the Super-SBM model to assess China's agricultural ecological efficiency from 2010 to 2020, a discernible trend emerges from Fig. 3: China's aggregate agricultural ecological efficiency has generally trended upwards. Despite a minor dip in 2017, the pace of this increase has subsequently quickened. This can be largely attributed to the Chinese government's policy initiatives during the 13th Five-Year Plan period, which were designed to safeguard farmland and bolster agriculture. These policies, steering towards green and ecological guidance, aimed to deepen the agricultural supply-side structural reform and hasten the modernization of both agriculture and rural regions. While these policies have significantly contributed to enhancing agricultural ecological efficiency, it is evident from an absolute value perspective that China's agricultural ecological efficiency remains in an early stage of development. Thus, there is a palpable need to delve deeper into both internal factors and external environmental factors to further enhance agricultural ecological efficiency.

#### 4.1.3 Interprovincial cluster analysis of the agricultural ecological efficiency in China

**Fig. 4:**
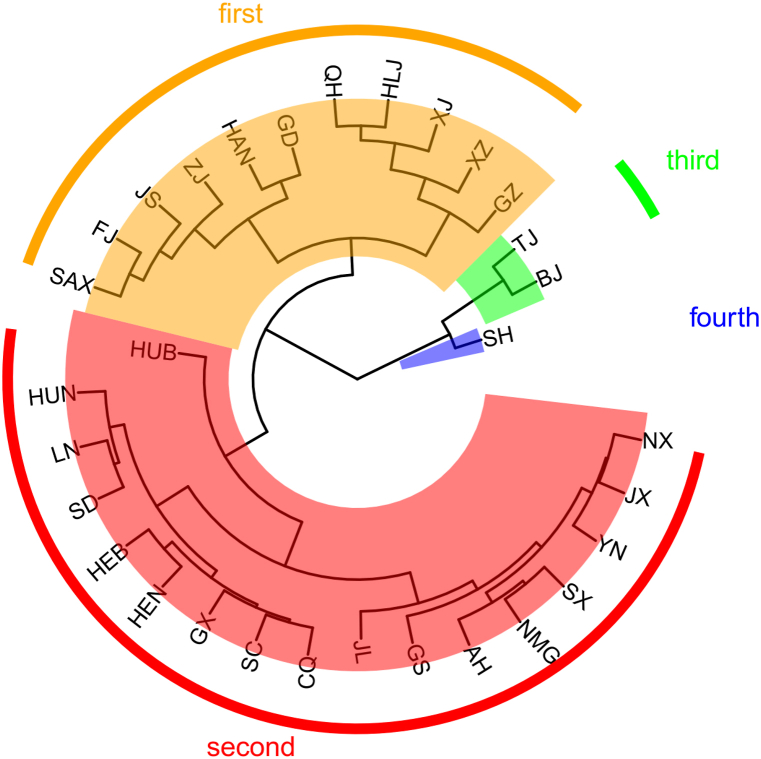
Clustering results of 31 provinces in mainland China based on agroecological efficiency.

**Fig. 5:**
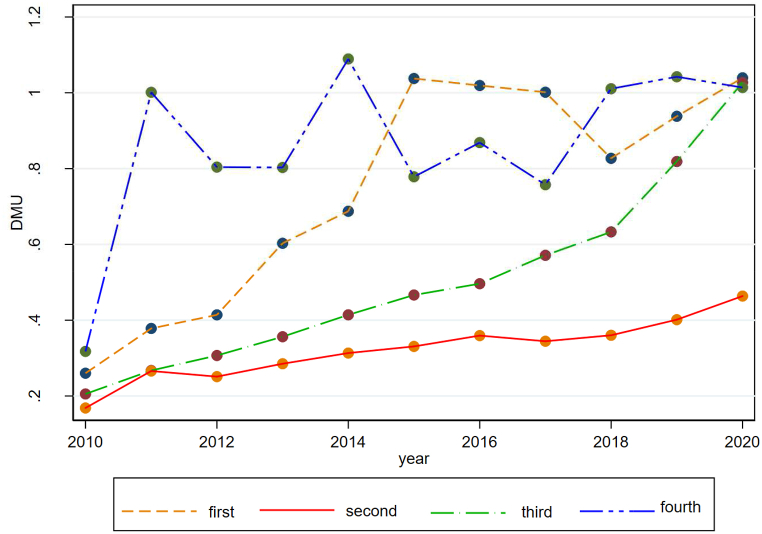
Trends of agricultural eco-efficiency in each group under clustering groups during 2010–2020.

Although the overall agricultural ecological efficiency in China has been on an upward trajectory, a divergent performance is observed among individual provinces. The 31 mainland Chinese provinces, are divided into four groups (Fig. 4), and the average trend of each from 2010 to 2020 is depicted in Fig. 5.

The first group, which includes provinces like Guangdong(GD), Fujian(FJ), and Hainan(HAN), demonstrated a swift rise in efficiency followed by sustained stability. Conversely, the second group, comprising 27 provinces—including Chongqing(CQ) and Hebei(HEB)—exhibited a persistently low-efficiency level. The third group, notably Beijing(BJ) and Tianjin(TJ), revealed an upward-trending index. Meanwhile, the fourth group, with Shanghai(SH) as a prime example, consistently manifested significant fluctuations over the past decade, yet displayed a slow increase.

These divergent patterns among the groups suggest that the enhancements in agricultural ecological efficiency across different regions over the past decade are potentially influenced by various factors, not confined merely to the spectrum of local economic development. For instance, the second group remains at a low-efficiency level despite comprising provinces with disparate economic statuses, indicating that aspects other than economic development could be influential. This variability across different city groups with distinct economic standings alludes to the plausible influence of other elements, such as local agricultural support policies and governance mechanisms, in shaping the agricultural ecological efficiency in each province. Therefore, it can be tentatively hypothesized that the variation is not solely tethered to local economic development, inviting further exploration into the multifaceted determinants impacting agricultural ecological efficiency across provinces.

### 4.2 Spatial association analysis

**Fig. 6:**
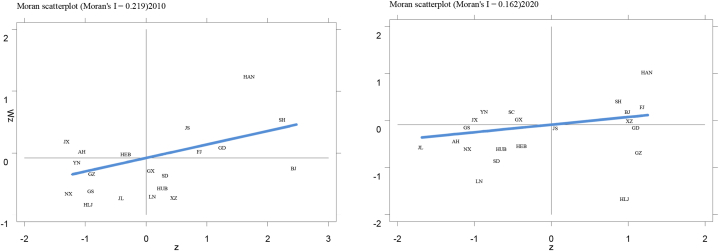
Scatter plot of local Moreland index of agricultural eco-efficiency in China in 2010 and 2020.

In order to delve into the contributing factors of agricultural ecological efficiency in China via a spatial panel model, an initial assessment of the spatial autocorrelation of the nation's agricultural ecological efficiency is imperative. The research employs Global Moran's I and Local Moran's I for this examination, with an exhaustive presentation of the test findings available in Table 3 and Fig. 6.

**Table 3:** Global Moran's I values of agricultural eco-efficiency in China during 2010–2020.

Year	Moran's I	P value
2010	0.219**	0.016
2011	0.001	0.368
2012	0.173**	0.017
2013	0.202**	0.016
2014	0.230***	0.006
2015	0.385***	0.000
2016	0.354***	0.000
2017	0.323***	0.001
2018	0.267***	0.005
2019	0.165**	0.049
2020	0.162**	0.054

Note: *** means significant at the significance level of 0.01, ** means significant at the significance level of 0.05, * means significant at the significance level of 0.1.

#### 4.2.1 Global Moran index

The global Moran index of China's agricultural ecological efficiency from 2010 to 2020 is calculated by using the number of adjacent borders of each province as the spatial weight matrix; for methodology details, refer to Młodak [38]and Anselin [39]. The results in Table 3 show that, except for 2011, the Moran's I values from 2010 to 2020 were uniformly positive, signaling a notable occurrence of agricultural ecological efficiency in recent years. Notably, there was a gradual decline in Moran's I value post-2015, signifying a progressive reduction in the spatial disparities of agricultural ecological efficiency within China. This trend coincides with China's intensified efforts toward rural revitalization and the rapid modernization of agriculture and rural areas. Consequently, there has been a surge in the cross-regional mobility of agricultural production factors and enhanced promotion of agricultural technology nationwide. Moreover, the frequent online trade of agricultural products across provinces and the strengthened agricultural cooperative activities among the provinces have bolstered the positive spillover effect of agricultural ecological efficiency.

#### 4.2.2 Local Moran index

The Local Moran's I index serves as an extension of the spatial analysis, providing more nuanced insights into the spatial clustering phenomena at the provincial level. To elucidate the disparities in agricultural ecological efficiency among various provinces, this study calculated the Local Moran's I for each province from 2010 to 2020. However, due to space limitations, scatter plots are only presented for the years 2010 and 2020, as shown in Fig. 6. For the local Moran index measurement method and scatter chart practices, readers are referred to the work of Kozonogova and Dubrovskaya [40]. Regarding the change in the quadrant distribution in the scatter map of the local Moran index in the 31 Chinese mainland provinces, several provinces transitioned from “low-low” clusters to “high-high” clusters, indicating an increase in provinces within the “high-high” category. Concurrently, there was a noticeable reduction in provinces categorized within the “high-low” clusters. These observations underscore the influential role of provinces with advanced agricultural ecological efficiencies. Through spatial spillover effects, these provinces appear to catalyze agricultural advancement in their neighboring regions.

### 4.3 Analysis of influencing factors of spatial and temporal variation in agricultural ecological efficiency in China

The agricultural ecological efficiency values measured by the traditional DEA model typically range from 0 to 1, and previous studies predominantly employ restricted dependent variable regression models to investigate the determinants of agricultural ecological efficiency within China [32,41]. While these models effectively mitigate the bias introduced by the non-negativity constraint in OLS estimates, they fall short in capturing the spatial spillover effects, a critical aspect of understanding the nuances of regional influences on regression outcomes. Addressing this gap, this paper adopts a spatial panel model approach, enabling a more nuanced analysis of the causal dynamics between agricultural ecological efficiency and the level of digitalization in China's agriculture sector, while providing an intricate decomposition of the impacts exerted by various contributing factors.

#### 4.3.1 Spatial panel model test

The determination of an appropriate model for analyzing the factors influencing agricultural ecological efficiency in China necessitates the utilization of a spatial panel model test. This process unfolds in several steps: initially, the Lagrangian Multiplier (LM) and the Robust Lagrangian Multiplier (RLM) are used to judge the model form of the spatial panel (whether the spatial correlation has a lag term or an error term). Subsequently, either Walds or likelihood ratio (LR) is used to test whether the Spatial Panel Durbin Model (SPDM) can degenerate into SPLM and SPEM. Table 4 illustrates the test results of the LM and RLM. From the significance of the test statistics, the LM of the SPEM and both the LM and the RLM of the SPLM are significant at the 0.01 level, while the RLM for the SPEM is not. This suggests that the influence of explanatory variables, notably the level of agricultural digitization, on agricultural ecological efficiency, is marked by spatial causality. Consequently, the application of the SPLM is deemed more appropriate than that of the SPEM.

**Table 4:** Correlation test results of spatial panel model.

Test Method	Statistic Value	P Value
LM error	26.505***	0.000
LM lag	33.980***	0.000
RLM error	0.021	0.885
RLM lag	7.495***	0.006

#### 4.3.2 Model comparison and selection

This study employs the Spatial Panel Durbin Model (SPDM) and compares it with the Spatial Panel Lag Model (SPLM) and the Spatial Panel Error Model (SPEM), with key test outcomes detailed in Table 5. The Hausman test statistics underscore the superiority of the random-effects over the fixed-effects formulation for the SPLM, while the fixed-effects versions are more fitting for both the SPEM and SPDM. Both the dispersion (Sigma^2^) and R^2^ metrics affirm the SPDM's predominance over the SPLM and SPEM. Furthermore, the LR test, significant at the 10% level, precludes the simplification of the SPDM into either the SPLM or SPEM, thereby validating the SPDM's optimal conformity in appraising the determinants of agricultural ecological efficiency in China. Column (3) of Table 5 exhibits the regression results of the spatial panel Durbin model (SPDM). The core explanatory variable, namely, the agricultural digital technology regression coefficient, is 0.3956, which is significantly positive at the 0.05 level, agro-digitization technology has a significant positive impact on agro-ecological efficiency, hypothesis H1 is proved. Furthermore, its corresponding spatial coefficient is significantly positive at the 0.1 level, which is 0.4866, denoting that agricultural digitalization contributes positively not just to the ecological efficiency of agriculture in the focal province, but also engenders beneficial spillover effects for adjacent provinces, hypothesis H2 is proved.

**Table 5:** Selection and comparison of SPLM, SPEM, and SPDM models.

Variables	SPLM （1）	SPEM （2）	SPDM （3）
LNDIGI	0.2928**(2.29)	0.3926**(2.08)	0.3956**(2.10)
LNGDP	0.0396(1.41)	0.3800***(7.92)	0.3056***(3.35)
LNFINA	1.4402***(2.94)	1.5210**(2.44)	1.2481*(1.91)
LNPOINC	−0.6758***(-4.66)	−0.3660*(-1.69)	−0.1959(-0.88)
LNAGMD	0.0290(0.59)	−0.0648(-0.90)	−0.0632(-0.88)
LNAGDR	−0.2081***(-2.66)	−0.1120(-1.42)	−0.0772(-0.98)
W* LNDIGI			0.4866*(1.91)
Control Variables	–	–	Spatial Lag
Sigma^2^	0.0156***	0.0138***	0.0133***
R^2^	0.4584	0.1237	0.4815
Hausman	−787.18***	65.53***	57.46***
LR Test	11.04*	14.25*	

#### 4.3.3 The spatial effect decomposition of each factor

To delve deeper into the marginal effects of agricultural digitalization on agricultural ecological efficiency in China, this study decomposes the spatial spillover effect, utilizing the methodology proposed by Lesage et al. to reveal the direct and indirect effects of the core explanatory variables, along with other control variables, on the agricultural ecological efficiency in both the province in question and its neighboring provinces. The results of this decomposition are presented in Table 6.

(1) Agricultural digitalization significantly boosts agricultural ecological efficiency, with both direct and indirect effects being notably positive. This underscores the importance of digitalization in enhancing agricultural ecological efficiency within provinces and across neighboring regions. At the production level, the integration of digital technologies, including the Internet of Things, Big Data, Artificial Intelligence, and other next-generation information technologies, into farming practices allows for refined crop cultivation strategies, precise sowing, accurate application of treatments, and real-time monitoring of crop growth, leading to substantial improvements in agricultural output. On the consumer side, rural online businesses, online shops, network broadcasts, net red county magistrates, and digital platforms facilitate diverse sales avenues for agricultural products, enabling urban dwellers to access rural commodities from afar. Additionally, digital platforms expand market reach for produce, allowing consumers to purchase specialty goods from remote rural areas. Further, grassroots digital strategies and macro-level policies are bridging primary, secondary, and tertiary sectors, ensuring agriculture's sustainable future. Digital technologies are also pivotal in transforming lifestyles and cutting carbon footprints across the agricultural supply chain. These effects not only elevate the agricultural ecological efficiency of individual provinces but also benefit the agricultural ecological efficiency of neighboring provinces through the “siphon effect” and “demonstration effect".
(2) Regarding other determinants of agricultural ecological efficiency, financial backing for agriculture has emerged as a positive influencer. Its direct and indirect impacts are noteworthy, indicating its pivotal role in fostering agricultural ecological efficiency locally and in adjacent provinces. This is attributed to China's nationwide financial initiatives targeting under-resourced rural areas. Substantial investments in rural revitalization and agricultural modernization have yielded improvements in both the ecological landscape and farmers' earnings, facilitating regional resource allocation and bolstering economic stature, which enhances agricultural conditions in adjacent areas. This pathway is further substantiated by the positive correlation between regional economic growth and agricultural ecological efficiency. Tests indicate that local economic advancements have a tangible impact on agricultural ecological efficiency, mirroring nationwide efforts to deepen industrial integration and service-oriented agricultural support, and bridging urban-rural disparities in income, healthcare, education, and infrastructure. However, data suggest that increased non-agricultural income among rural households does not dampen agricultural production enthusiasm or ecological efficiency directly. Conversely, the indirect effect is markedly positive, indicating that heightened non-agricultural income potentially boosts demand for goods from neighboring provinces, thereby enhancing their agricultural ecological efficiency through spatial interconnectedness.

**Table 6:** Direct, indirect, and total effects of factors influencing agricultural eco-efficiency in China.

Variables	Direct effect （1）	Indirect effect （2）	Total effect （3）
LNDIGI	0.4399**(2.26)	0.7546**(2.30)	1.1945***(2.92)
LNGDP	0.3102*** (3.70)	0.1646 (1.31)	0.4749***(4.86)
LNFINA	1.4908**(2.47)	3.4759***(2.86)	4.9668***(3.97)
LNPOINC	−0.1413(-0.65)	1.1126***(2.86)	0.9713*(1.79)
LNAGMD	−0.0570(-0.81)	0.0788(0.48)	0.0218(0.12)
LNAGDR	−0.0686 (−0.89)	0.1083(-1.42)	0.0397(0.19)

#### 4.3.4 Robustness analysis

(1) Utilizing geographic weighting matrices to replace 0–1 matrices. To ensure the reliability of the spatial measurement model, the distance between the provinces was calculated, and the geographic weight matrix was calculated. First, the matrix was employed to estimate the coefficients within the spatial Durbin model, followed by an effect decomposition. The results are shown in Table 7. Within the 0-1 adjacency weight matrix, the positive and negative signs and the significance of the agricultural digitization level of the core explanatory variable remain unaltered, indicating that the research conclusions of this paper are reliable.

**Table 7:** Direct, indirect, and total effects of factors influencing agricultural eco-efficiency in China based on geographical weight matrix.

Variables	Direct effect	Indirect effect	Total effect
LNDIGI	0.3692**(2.45)	0.6325**(2.62)	1.0017***(2.86)
LNGDP	0.3252*** (3.78)	0.2251* (1.87)	0.5403***(5.18)
LNFINA	1.5018**(2.51)	3.6658***(2.91)	5.1676***(4.26)
LNPOINC	0.2516(1.65)	1.5627***(2.98)	1.8143***(2.45)
LNAGMD	−0.0680(-0.88)	0.0872(0.48)	0.0192(0.11)
LNAGDR	−0.0268 (−0.46)	0.2132(-1.62)	0.1864(1.23)

(2) Instrumental variable approach. This paper focuses on the impact of the core explanatory variable, agricultural digitization level, on agroecological efficiency in China. Generally speaking, the higher the agroecological efficiency of a region, the more likely it is that investment in agricultural digitization construction will increase the level of agricultural digitization in the area. Thus, the level of agricultural digitization and agroecological efficiency may have a causal relationship. Additionally, agroecological efficiency is influenced not only by macroeconomic factors but also by micro-factors such as the customs and personal characteristics of the region. However, these factors are not accommodated within the model due to the limitation of data availability. The preceding fixed-effects model partially mitigates the concern of omitted variables. To address the impact of endogenous explanatory variables on the model's robustness, this paper employs the panel instrumental variable method to reinforce the validity of the regression results of the model (2). In the choice of instrumental variables, this paper opts for DIGIF, defined as the word frequency of digital economy policy mentioned in regional governmental work reports, serving as an instrumental variable for the level of agricultural digitization. This is predicated on the observation that a higher frequency of digital economy terms in governmental reports usually correlates with a more advanced local digital landscape. Nonetheless, within the intrinsic rationale of these reports, the word frequency of digital economy terms does not exhibit a significant connection with local agricultural ecological efficiency. The Two-Stage Least Squares (2SLS) method was utilized to identify the causality of the relationship between the level of agricultural digitization and agroecological efficiency, with the estimation findings presented in Table 8.

**Table 8:** Results of instrumental variables on the impact of the level of agricultural digitization on agroecological efficiency.

	（1）	（2）
	first stage	second stage
Variables	LNDIGI	DMU
DIGIF	0.0689***	
	(0.0116)	
LNDIGI		0.7069***
		(0.1226)
Constant	−0.2544**	1.2571***
	(0.1274)	(0.2593)
Control variable	Yes	Yes
Area fixed effect	Yes	Yes
Year fixed effects	Yes	Yes
Observations	330	330
R-squared	0.635	0.620

***p < 0.01, **p < 0.05, *p < 0.1.

From the regression results, the regression coefficients of both stages are significantly positive, revealing that the positive correlation between the level of agricultural digitization and agroecological efficiency still holds significantly after the resolution of endogeneity.

## 5 Conclusions

This paper utilizes agricultural input and output panel data from various provinces and cities in China, employing the Super-SBM model to calculate the agroecological efficiency of 31 provinces in mainland China from 2010 to 2020. It then uses the spatial Durbin model, with the level of agricultural digitalization as the core explanatory variable, to examine the spillover effects of influencing factors. Compared to previous literature, this study not only measures the spatial disparities and correlations of agroecological efficiency in China but also focuses on analyzing the impact pathway and spatial spillover effects of agricultural digital technology on agroecological efficiency, yielding the following conclusions.(1)In China's agricultural production inputs, investments in fixed assets and land have shown a steady upward trend, while inputs of pesticides and fertilizers have decreased annually after 2015. This indicates a gradual shift from labor-intensive and environmentally harmful resource factors to socio-economic and eco-friendly elements under the “dual carbon” goal and new urbanization requirements.(2)The agroecological efficiency in China overall presents a linear upward trend over time, but the efficiency values remain lower compared to developed countries.(3)Spatially, agroecological efficiency in China exhibits agglomeration and heterogeneity, with the agglomeration degree and level values in eastern provinces significantly higher than those in central and western regions. Agroecological efficiency is correlated not only with local economic development but also with regional economic development levels.(4)The level of agricultural digitalization has a significant positive direct effect and spatial spillover effect on agroecological efficiency, with the spatial spillover effect larger in magnitude than the direct effect.(5)Among the control variables, financial support for agriculture and local economic development have positive direct effects on local agroecological efficiency, while the proportion of rural household operating income has a positive spatial spillover effect on adjacent areas.

The conclusions provide substantial policy insights.(1)In the context of rural revitalization in China, emphasis should be placed on transitioning agricultural development from quantity to quality, increasing investment in agricultural technology, and reducing inputs of traditional polluting production factors. (2)Accelerate digital rural construction, leverage fiscal investments in digital infrastructure and platform building, and enhance the application level of digital technology in agricultural production, exchange, and consumption.(3)Facilitate the circulation and collaboration of digital resources across regions, increase the number of agricultural digital technology application service enterprises, concentrate efforts on developing modern agricultural technologies like big data, cloud computing, and blockchain, and achieve effective integration of primary, secondary, and tertiary industries.(4)Strengthen regional cooperation, formulate policies to promote the flow of talent, capital, and technology to areas with lower agroecological efficiency, emphasize the effective combination of agricultural resource endowments with modern technology, utilize regional resource advantages fully, and develop large-scale, characteristic agricultural industry systems to enhance the quality of agricultural supply and output efficiency.(5)Enhance supervision of the land management process to prevent falling into the “profit-seeking trap” and eliminate inefficient agricultural income gained at the expense of the agricultural ecological environment.

Although China's agricultural ecological efficiency has improved significantly under agricultural modernization and management system reforms, questions remain on how to implement diverse agricultural planting structures and production and management practices based on different agricultural resource characteristics, how to devise incentives to attract high-skilled digital technology talents to agriculture, and how to implement distribution systems that ensure the benefits of improved agroecological efficiency are shared among a broader group. These issues warrant attention in practice and merit further exploration.

## Data availability statement

Data will be made available on request, further inquiries can be directed to the corresponding author.

## CRediT authorship contribution statement

**Haoran Wang:** Writing – review & editing, Writing – original draft, Validation, Methodology, Investigation. **Zhuangzhuang Li:** Visualization, Supervision, Software, Resources, Project administration, Methodology, Funding acquisition, Formal analysis, Data curation, Conceptualization.

## Declaration of competing interest

The authors declare that they have no known competing financial interests or personal relationships that could have appeared to influence the work reported in this paper.

## Footnotes

1 The 31 provinces in mainland China: Beijing(BJ),Tianjin(TJ),Hebei(HEB),Shanxi(SX), Neimenggu(NMG),Liaoning(LN),Jilin(JL),Heilongjiang(HLJ),Shanghai(SH),Jiangsu(JS),Zhejiang(ZJ),Anhui(AH),Fujian(FJ), Jiangxi(JX),Shandong(SD),Henan(HEN),Hubei(HUB), Hunan(HUN), Guangdong(GD), Guangxi(GX),Hainan(HAN),Chongqing(CQ), Sichuan(SC),Guizhou(GZ),Yunnan(YN),Xizang(XZ),Shaanxi(SAX),Gansu(GS),Qinghai(QH),Ningxia(NX),Xinjiang(XJ).
